# Do the Preferences of Healthcare Provider Selection Vary among Rural and Urban Patients with Different Income and Cause Different Outcome?

**DOI:** 10.1371/journal.pone.0152776

**Published:** 2016-04-07

**Authors:** Tsung-Hsien Yu, Kuo-Piao Chung, Chung-Jen Wei, Kuo-Liong Chien, Yu-Chang Hou

**Affiliations:** 1 Institute of Health Policy and Management, National Taiwan University, Taipei, Taiwan; 2 Department of Public Health, Fu-Jen Catholic University, Taipei, Taiwan; 3 Institute of Epidemiology and Preventive Medicine, National Taiwan University, Taipei, Taiwan; 4 Department of Chinese Medicine, Tao-Yuan General Hospital, Ministry of Health and Welfare, Taoyuan, Taiwan; 5 Department of Bioscience Technology, Chuan-Yuan Christian University, Taoyuan, Taiwan; Sichuan University, CHINA

## Abstract

**Background:**

Equal access to healthcare facilities and high-level quality of care are important strategies to eliminate the disparity in outcome of care. However, the existing literature regarding how urban or rural dwelling patients with different income level select healthcare providers is insufficient. The purposes of this study were to examine whether differences of healthcare provider selection exist among urban and rural coronary artery bypass surgery (CABG) patients with different income level. If so, we further investigated the associated impact on mortality.

**Methods:**

A retrospective, multilevel study design was conducted using claims data from 2007–2011 Taiwan’s Universal Health Insurance Scheme. Healthcare providers’ performance and patients’ travelling distance to hospitals were used to define the patterns of healthcare provider selection. Baron and Kenny’s procedures for mediation effect were conducted.

**Results:**

There were 10,108 CABG surgeries included in this study. The results showed that urban dwelling and higher income patients were prone to receive care from better-performance providers. The travelling distances of urban dwelling patients was 15 KM shorter, especially when they received better-performance provider’s care. The results also showed that the difference of healthcare provider selection and mortality rate existed between rural and urban dwelling patients with different income levels. After the procedure of mediation effect testing, the results showed that the healthcare provider selection partially mediated the relationships between patients’ residential areas with different income levels and 30-day mortality.

**Conclusion:**

Preferences of healthcare provider selection vary among rural and urban patients with different income, and such differences partially mediated the outcome of care. Health authorities should pay attention to this issue, and propose appropriate solutions to eliminate the disparity in outcome of CABG care.

## Introduction

Equal access to health facilities and to ensure an equal level of high-quality care for all patients who present to the healthcare system with the same clinical indications regardless of race, ethnicity, gender, or socioeconomic status, are important strategies to eliminate the disparity in outcome of care in every country. [1] Differences in health outcomes between income levels and residence locations have been documented, [2–5] with the existing literature pointing out low-income or rural dwelling patients are more likely to receive sub-optimal care and worse outcome.[6, 7] However, the existing literature regarding how urban or rural dwelling patients with different income level select healthcare providers is insufficient.

In Taiwan, the National Health Insurance Scheme was established in 1995, and covers 99% of the population. People in Taiwan enjoy full accessibility to medical care; any barriers to health care have been reduced, and indeed, even no longer exist. [8] Although previous studies have demonstrated that the National Health Insurance Scheme has brought several positive effects on health, [9] health disparities among rural and urban dwellers still exist, [10] and this is worthy of in-depth investigation.

Coronary artery bypass surgery (CABG) is a high-risk surgery with mortality of around 5%. Many healthcare-related agencies and quality indicator projects selected this surgery to monitor the provider’s performance, e.g. OECD Health Care Quality Indicators project, Agency for Healthcare Research and Quality (AHRQ) in U.S. Therefore, the current study takes CABG as an example, to investigate whether differences of healthcare provider selection exist among urban and rural dwelling patients with different income level. If so, we further investigated whether the relationships between urban and rural dwelling patients with different income levels and mortality rates are mediated by such differences.

## Materials and Methods

### Study design

This retrospective and cross-sectional study adopted a multilevel design to examine the relationships between patient residence’s urbanization level with different income level, healthcare provider selection, and treatment outcomes among CABG patients after adjusting for patient-, surgeon-, and hospital-level covariates.

### Database

We used data from the Taiwan National Health Insurance Research Database (NHIRD) between 2007 and 2011. The NHIRD includes all the original outpatient, ambulatory and inpatient care claims data and registration files for beneficiaries enrolled under the NHI program. This database covers the 23 million enrollees in the NHI program (approximately 99% of Taiwan’s population). The NHI claims data provides de-identified, secondary patient-level demographic, administrative information and discharge status on every case, and this database can be accessed by the public for research purposes.

### Ethics Statement

The protocol for this study was approved by the Institutional Review Board of the National Taiwan University Hospital (protocol #201412074W). The dataset we used in this study was secondary data; all information was de-identified by data owners.

### Study population and Exclusion criteria

We restricted our analysis to hospitalization records in which patients had a procedure code indicating a CABG (ICD-9 CM procedure codes 36.1x–36.2x) [11] from January 1, 2008 to September 30, 2011. We excluded patients under the age of 18 years (n = 14) to restrict our evaluation to an adult population. Hospitalization records with missing data for gender (n = 3) were excluded. In addition, we also excluded patients who received surgeries from surgeons who never performed any CABG surgeries in the previous year (n = 27), for homogenizing the variation of surgeon’s performance.

### Definition of variables

#### Dependent variable: any cause 30-day mortality

The dependent variable in this study was 30-day mortality from any cause after hospitalization for CABG surgery; 30-day mortality was determined by linking inpatient admission records with the withdrawal certificate records. The only reason for being withdrawn from NHI coverage within 30 days of hospital admission would be death. Withdrawal dates are the same as the date deceased according to the death certificate. [12, 13]

#### Independent variable

*Urbanization level*: Patient’s residential areas were linked to the urbanization level. However, Taiwan’s NHI is an occupation-based social insurance scheme. Employees of large enterprises might be enrolled using the address of their company’s headquarters rather than the actual address of residence. Following Chang et al,[14] the actual location of each patient was assumed to be where an individual had the most outpatient and pharmacy visits in this study.

The location of each clinic and pharmacy was recognized as either urban or rural type according to the definition of urbanization published by Taiwan’s National Health Research Institutes. All 365 townships in Taiwan were classified into seven clusters based on the following indicators: Population density (people/km2), proportion of people with a college undergraduate degree or above, proportion of elder people over 65 years of age, proportion of people who were agriculture workers, and the number of physicians per 100,000 people. Residential areas located in clusters of 1 to 3 were categorized as urban, and the others as rural.[15]

*Income level*: Patients’ insurance identification records were used to distinguish patients in the low-income group from those who were not. In Taiwan, the National Health Insurance scheme classifies the insured into six insured classifications, according to the insured’s occupation. Households below the poverty line belong to classification 5. We used this information in NHIRD as a criterion to identify the income level. Furthermore, the low-income population accounted for 1% of the total population; therefore, we selected the top 1% of insured level (NTD 92,000/ USD 3,000) as the definition of high income. Lastly, we divided the remaining insured level in half- middle-high income (insured level is higher than NTD 28,000/ USD 900) and middle-low income.

#### Mediator Variable: healthcare provider selection

The definition of patterns of healthcare provider selection was the combination of the provider’s performance and distance to hospital. The definition of provider’s performance and distance to hospital are as below:

*Providers’ Performance*: The risk-adjusted 30-day mortality rates, risk-adjusted surgical site infection (SSI) rates, and service volumes of each hospital and for each surgeon in the previous year before each CABG surgery were used to evaluate the quality of CABG. Data on patient gender, age, Charlson/Romano Comorbidity Index (CCI) and number of vessels obstructed were incorporated for risk adjustment.

Nevertheless, too many indicators can make interpretation difficult. Therefore, a transformation algorithm was required to understand the meaning of quality indicators in a simple manner. In this study, we applied the k-means clustering algorithm to classify the quality of hospitals and surgeons in this study. K-means clustering algorithm was based on cluster analysis, it is a kind of data mining approach, and is also one of the most used methods for partitioning clusters. [16] We applied this approach in our previous work. [6, 7]

Surgeons and hospitals were assigned to “good performance” and “non-good performance” groups according to their distance to cluster centers. Patients who went to a “good performance” hospital and receive healthcare from a “good performance” surgeon were included in the “excellent care” group. If patients received care from a not good performance surgeon vs. a not good performance hospital, they would be included in the “not excellent care” group. The remainder (good performance surgeon vs. not good performance hospital, or not good performance surgeon vs. good performance hospital) were included in the “good care” group.

*Distance to hospital*: In term of distance, this study obtained the coordinates of the center of town where hospitals were located in or patients resided in through the Geographic Information System (ArcGIS for desktop, version 10.3), and calculated the Euclidean distance between these two points. The distances to hospitals were classified into near-, middle-, and far-distance groups, and 15 and 30 km were used as cutoff points according to travelling time (<30 minutes and > 1 hour).

*Healthcare Provider Selection*: After retrieving the information of providers’ performance and distances to hospital, this study used the combinations of providers’ performance and distances to hospital to produce nine patterns of healthcare provider selection; these were excellent performance-near distance, excellent performance-middle distance, excellent performance-far distance, good performance-near distance, good performance-middle distance, good performance-far distance, not good performance-near distance, not good performance-middle distance, and not good performance-far distance.

#### Covariates

In addition to three important patient-level variables we mentioned above, this study also collected other patient-, surgeon-, and hospital-level data. First, patient-level variables included age, gender, Charlson/Romano Comorbidity Index, and number of obstructed vessels (as a proxy indicator for duration of operation[17] that were involved in the surgical operation. Second, surgeon-level variables included age. Third, hospital-level variables included hospital ownership and accreditation status.

#### Statistical analysis

All statistical analyses were performed using SAS (version 9.4, SAS Institute Inc., Cary, NC, USA). In statistical testing, a two-sided p value ≤ 0.05 was considered statistically significant. The distributional properties of continuous variables were expressed by mean ± standard deviation (SD), and the categorical variables were presented by frequency and percentage. In bivariate analysis, potential predictors of 30-day mortality were examined using the chi-square test and the two-sample t-test as appropriate. To account for correlations of information within the healthcare provider, multivariable analysis was conducted by fitting multilevel or mixed-effects logistic/ multinomial logistic regression (for three levels of outcome variable) models to each patient’s data and then estimating the effects of hospital- and surgeon-level predictors on the probability of 30-day mortality. In addition, we combined Baron and Kenny’s mediation effect testing procedure [18] with the recommendations given by Mathieu *et al* [19] to examine the mediation effect among residential area with income level, healthcare provider selection, and 30-day mortality. Finally, Sobel’s test was used to verify the significance of the mediation test.[20]

#### Sensitivity analysis

The cutoff values of travelling distance were determined in subjective manner; this study also categorized it into three groups again by k-means clustering algorithm for sensitivity analysis.

## Results

There were 10,108 CABG operations performed by 317 surgeons in 60 hospitals from January 1, 2008 to September 30, 2011 that were included. Table 1 demonstrates the results of descriptive analysis. Among these cases, 4,778 (47.27%) patients lived in urban areas, and the rest of them lived in rural areas. Around 70% of the studied patients received their surgeries in medical centers. Thirty-six percent of the patients went to a public hospital; the average hospital service volume, risk-adjusted SSI rates and risk-adjusted 30-day mortality rates were 147, 1.29% and 5.42% respectively. With respect to surgeon characteristics, the mean age of these surgeons being 44 years, the average surgeon service volume, risk-adjusted SSI rates and risk-adjusted 30-day mortality rates were 50.30, 1.27% and 4.92% respectively. Around one-fourth of the patients were female, and their mean age was 65 years. Around 60% of patients had more than two vessels being obstructed. One-fourth of the patients were classified as high or middle-high income, and half of them received excellent or good care; most patients selected the hospital nearby their residence location, the average travelling distance to hospital was 36 kilometers, and 584 patients (5.78%) died within 30 days after hospitalization.

**Table 1:** Descriptive Analysis.

	All	Resident Areas		*p*-value
		Urban Areas(*n* = 4,778)	Rural Areas(*n* = 5,330)	
**Hospital-level**				
Accreditation status, *n* (%)				< .0001^†^
Medical center	6,821(67.48)	3,485(72.94)	3,336(62.59)	
Not medical center	3,287(32.52)	1,293(27.06)	1,994(37.41)	
Ownership, *n* (%)				0.0098^†^
Public hospital	3,632(35.93)	1,779(37.23)	1,853(34.77)	
Not public hospital	6,476(64.07)	2,999(62.77)	3,477(65.23)	
Hospital service volume, mean (S.D.)	147.22(92.25)	159.6(89.89)	136.2(92.94)	< .0001^‡^
Hospital risk-adjusted infection rate (%), mean (S.D.)	1.29(1.70)	1.35(1.63)	1.23(1.76)	0.0004^‡^
Hospital risk-adjusted 30-day mortality rate (%), mean (S.D.)	5.42(5.46)	5.31(4.76)	5.52(6.02)	0.0543^‡^
**Surgeon-level**				
Surgeon’s age, mean (S.D.)	44.44(7.63)	44.53(7.66)	44.36 (7.59)	0.2653^‡^
Surgeon service volume, mean (S.D.)	50.30(34.72)	53.42(35.82)	47.50 (33.46)	< .0001^‡^
Surgeon risk-adjusted infection rate (%), mean (S.D.)	1.27(3.11)	1.35(3.55)	1.19 (2.65)	0.0109^‡^
Surgeon risk-adjusted 30-day mortality rate (%), mean (S.D.)	4.92(7.51)	4.87(6.79)	4.96(8.11)	0.5480^‡^
**Patient-level**				
Age, mean (S.D.)	65.66(11.16)	65.17(11.32)	66.11 (11.00)	< .0001^‡^
Gender, *n* (%)				0.0001^†^
Female	2,376(23.51)	1,040(21.77)	1,336(25.07)	
Male	7,732(76.49)	3,738(78.23)	3,994(74.93)	
CCI, *n* (%)				0.0018^†^
< = 1	5,341(52.84)	2,603(54.48)	2,738(51.37)	
2+	4,767(47.16)	2,175(45.52)	2,592(48.63)	
Number of vessels obstructed, *n* (%)				0.0256^†^
1	4,221(41.76)	1,940(40.60)	2,281(42.80)	
2+	5,887(58.24)	2,838(59.40)	3,049(57.20)	
Income level				< .0001^†^
High and middle-high	2,588(25.60)	1,554(32.52)	1,034(19.40)	
Low and middle-low	7,520(74.40)	3,224(67.48)	4,296(80.60)	
Provider’s performance				< .0001^†^
Excellent	2531(25.04)	1,378(28.84)	1,153(21.63)	
Good	2924(28.93)	1,427(29.87)	1,497(28.09)	
Not-good	4653(46.03)	1,973(41.29)	2,680(50.28)	
Travelling distances to hospital, mean (S.D.), kilometer	35.93(60.52)	27.98(58.21)	43.05 (61.66)	< .0001^‡^
Travelling distances to hospital				< .0001^†^
Near-distance	4,093(40.49)	2,496(52.24)	1,597(29.96)	
Middle-distance	3,359(33.23)	1,596(33.40)	1,763(33.08)	
Far-distance	2,656(26.28)	686(14.36)	1,970(36.96)	
30-day mortality, *n* (%)	584(5.78)	213(4.46)	371(6.96)	< .0001^†^

^†^ χ^2^ test

^‡^*t*-test

The data also showed that rural-dwelling patients were poorer, older and were more likely to have comorbidity issues. The results also revealed that the percentage of rural dwelling patients who received care with excellent quality was lower than that of urban dwelling patients (21.63% vs. 28.84%), but the percentage of rural dwelling patients who received care from provider with not good performance was higher (41.29% vs. 50.28%). The travelling distance to hospital of urban dwelling patients was shorter than that for rural dwelling patients. The 30-day mortality was found to be higher in rural dwelling patients (6.96% vs. 4.46%). Besides, the urban dwelling patients were prone to receive surgeries in medical centers and public hospitals than were rural dwelling patients. Regarding hospital performance, hospitals visited by patients from rural areas had lower service volumes (136 vs. 160) and similar risk-adjusted 30-day mortality rates (5.52% vs. 5.31%). However, these hospitals had better risk-adjusted SSI rates than those visited by urban dwelling patients (1.23% vs. 1.35%). Regarding surgeon performance, surgeons who served rural dwelling patients had lower service volumes (48 vs. 53) but better risk-adjusted SSI rates (1.19% vs. 1.35%). The surgeon-level risk-adjusted 30-day mortality rates were similar between surgeons serving the two patient groups.

Table 2 shows that the distribution of patterns of healthcare provider selection. More than 50% urban dwelling patients went to the hospitals nearby their residence location, no matter what income level they had. However, the percentage of rural dwelling patients who went to the hospitals nearby their residence location was only around 30%. Besides, urban dwelling patients who went to the nearby hospital had a higher percentage of receiving higher performance provider’s surgery than did rural dwelling patients, no matter what income level they had. Moreover, the differences of patterns of healthcare provider selection between different income levels among urban dwelling patients were quite similar than rural dwelling patients. Rural dwelling patients with lower income seemed to select a worse pattern of healthcare provider selection. (i.e. longer travelling distance and poorer provider’s performance)

**Table 2:** Distribution of patterns of healthcare provider selection: stratified by residence area and income level.

	Urban				Rural				p-value^¶^
	All	E-P	G-P	NE-P	All	E-P	G-P	NE-P	
High and middle-high income									
N-D	782	232(29.67)	250(31.97)	300(38.36)	344	58(16.86)	88(25.58)	198(57.56)	<0.0001
M-D	492	172(34.96)	123(25.00)	197(40.04)	331	97(29.31)	96(29.00)	138(41.69)	0.1960
F-D	280	56(20.00)	76(27.14)	148(52.86)	359	122(33.98)	95(26.46)	142(39.55)	0.0002
Overall	1,554	460(29.60)	449(28.89)	645(41.51)	1,034	277(26.79)	279(26.98)	478(46.23)	0.0573
Low and middle-low income									
N-D	1,714	455(26.55)	507(29.58)	752(43.87)	1,253	168(13.41)	247(19.71)	838(66.88)	< .0001
M-D	1,104	343(31.07)	329(29.80)	432(39.13)	1,432	252(17.60)	438(30.59)	742(51.82)	< .0001
F-D	406	120(29.56)	142(34.98)	144(35.47)	1,611	456(28.31)	533(33.09)	622(38.61)	0.5049
Overall	3,224	918(28.47)	978(30.33)	1328(41.19)	4,296	876(20.39)	1218(28.35)	2202(51.26)	< .0001

^¶^χ^2^ test

E-P: Excellent performance; G-P: Good performance; NE-P: Not excellent performance

N-D: Near distance; M-D: Middle distance; F-D: Far distance

Table 3 demonstrates the travelling distance to different provider’s performance level among rural and urban dwelling patients with different income levels. In general, the travelling distances of urban dwelling patients was shorter than rural dwelling patients. The results also revealed that the rural dwelling patients needed to move farther, then received care from excellent performance provider, especially in patients with low and middle-low income levels.

**Table 3:** Travelling distance to different level of provider performance: stratified by residence area and income level.

	Urban	Rural	Difference
High and middle-high income			
Excellent performance	27.03(57.73)	54.31(69.94)	27.28(62.59)***
Good performance	35.13(71.10)	37.57(55.37)	2.44 (65.53)
Not excellent performance	45.11(80.14)	43.29(70.54)	-1.83(76.20)
Low and middle-low income			
Excellent performance	23.55(50.78)	62.99(75.27)	39.44(63.92) ***
Good performance	25.15(50.63)	45.78(59.79)	20.63(55.89) ***
Not excellent performance	22.72(48.43)	32.82(51.10)	10.11(50.11) ***

Mean (SD)

***<0.001

Table 4 demonstrates the mortality rate among rural and urban dwelling patients with different income levels in different patterns of healthcare provider selection. The results showed that the difference of mortality rate existed between rural and urban dwelling patients with different income levels. The results also revealed that the patterns of healthcare provider selection might cause the mortality difference between urban and rural dwelling patients with lower income, especially in patients with low and middle-low income who selected hospitals close by.

**Table 4:** Mortality rate among patterns of healthcare provider selection stratified by residence area and income level.

	Urban			Rural			p-value†
	E-P	G-P	NE-P	E-P	G-P	NE-P	
**High and middle-high income**							
Near distance	2(0.86)	8(3.20)	19(6.33)	1(1.72)	5(5.68)	19(9.6)	0.0811
Middle distance	0(0.00)	4(3.25)	14(7.11)	3(3.09)	5(5.21)	8(5.80)	0.4945
Far distance	0(0.00)	1(1.32)	6(4.05)	3(2.46)	7(7.37)	9(6.34)	0.0423
**Low and middle-low income**							
Near distance	7(1.54)	16(3.16)	54(7.18)	9(5.36)	19(7.69)	96(11.46)	< .0001
Middle distance	9(2.62)	17(5.17)	34(7.87)	15(5.95)	21(4.79)	73(9.84)	0.1193
Far distance	1(0.83)	11(7.75)	10(6.94)	9(1.97)	17(3.19)	52(8.36)	0.5377

E-P: Excellent performance; G-P: Good performance; NE-P: Not excellent performance; n(%)

† logistic regression

Table 5 shows the preferences of patients’ selection after adjusting covariates. In terms of level of provider’s performance, low and middle-income patients who lived in rural area were prone to receive care from provider with poorer performance, comparing with high and middle-high income patients in urban areas, after adjusting covariates. On the other hand, in terms of travelling distance, the rural dwelling patients were prone to move a further distance to receive care than high and middle-high income patients in urban areas. However, the results also demonstrated the urban dwelling patients with low and middle-low income were prone to stay in their local area to receive their care than were high and middle-high income patients in urban areas.

**Table 5:** Preferences of patients’ selection.

	Level of Provider’s Performance (Ref = excellent)			Travelling distance (Ref = Near)		
	Odds ratio	95% C.I.		Odds ratio	95% C.I.	
		LCL	UCL		LCL	UCL
Residential Area(ref. = Urban/ HMI)						
Rural/ LMI	0.685	0.582	0.806	0.386	0.329	0.454
Urban/ LMI	0.987	0.837	1.164	1.211	1.033	1.419
Rural/ HMI	0.866	0.696	1.077	0.454	0.369	0.558

HMI: High or middle-high income; LMI: Low or middle-low income

Adjusted by hospital accreditation status, ownership, surgeon’s age, patient’s gender, age, number of vessels obstructed, and comorbidity index.

C.I.: confidence interval; LCL: Lower confidence limit; UCL: Upper confidence limit

Table 6 demonstrates the results of mediation effect examination, using multilevel model. Model 1 aimed to verify any linkage between patient’s residential area with income level and 30-day mortality. The results suggested that rural dwelling patients were associated with a higher 30-day mortality risk (aOR = 1.512, 95% CI = 1.104–2.072; aOR = 1.826, 95% CI = 1.221–2.730) than were urban dwelling patients with higher income. Model 2 shows the relationship between patient’s residential areas with income level and patterns of healthcare provider selection. The results from this model indicated that rural dwelling patients were less likely to select better patterns of healthcare provider selection (aOR = 0.562, 95% CI = 0.504–0.626/β = -0.577, standard error = 0.055; aOR = 0.715, 95% CI = 0.623–0.819/β = -0.336, standard error = 0.070). Model 3 tested whether a mediation effect from patterns of healthcare provider selection existed within the relationship between patient’s residential area with income level and postoperative 30-day mortality. The results indicated that when patient’s residential area with income level and patterns of healthcare provider selection were both placed in the model, the rural dwelling patients who selected worse patterns of healthcare provider selection had higher mortality risk (aOR = 1.380, 95% CI = 1.007–1.894/ β = 0.322, standard error = 0.158; aOR = 1.734, 95% CI = 1.165–2.580/ β = 0.550, standard error = 0.202). Furthermore, the effect of patient’s residential area with income level on 30-day mortality was decreasing, from 1.512 to 1.380 in rural dwelling patients with lower income, and from 1.826 to 1.734 in rural dwelling patients with higher income. In this case, the t-value of Sobel’s test was -2.001 (t=(−0.577×0.322)(−0.577)2(0.158)2+(0.322)2(0.055)2=−2.001) in rural dwelling patient with lower income, and -2.368 (t=(−0.336×0.550)(−0.336)2(0.202)2+(0.550)2(0.070)2=−2.368) respectively. The result of Sobel’s test suggested a significant mediation effect, which meant the relationship between a patient’s residential area with income level and the 30-day mortality was partially mediated by patterns of healthcare provider selection.

**Table 6:** Results of Multilevel Analysis: Mediation Effect Examination.

	Model 1			Model 2			Model 3		
	Odds ratio	95% C.I.		Odds ratio	95% C.I.		Odds ratio	95% C.I.	
		LCL	UCL		LCL	UCL		LCL	UCL
Fixed-effects									
**Hospital-level**									
Accreditation status(ref. = Medical center)	1.536	1.082	2.181	0.780	0.598	1.018	1.344	0.955	1.892
Ownership(ref. = Public)	0.997	0.695	1.430	0.418	0.316	0.554	0.890	0.624	1.269
**Surgeon-level**									
Surgeon’s age	0.957	0.936	0.978	1.034	1.019	1.050	0.965	0.945	0.986
**Patient level**									
Residential Area(ref. = urban, HMI)									
Rural, LMI	1.512	1.104	2.072	0.562	0.504	0.626	1.380	1.011	1.883
Urban, LMI	1.154	0.831	1.605	1.066	0.961	1.183	1.170	0.845	1.619
Rural, HMI	1.826	1.221	2.730	0.715	0.623	0.819	1.734	1.165	2.580
Patterns of healthcare provider selection (ref. = excellent performance and short distance)							0.857	0.803	0.914
Gender(ref. = Male)	1.396	1.151	1.693	0.954	0.882	1.032	1.384	1.142	1.676
Age	1.051	1.041	1.062	0.999	0.996	1.002	1.052	1.042	1.062
Number of vessels obstructed (ref. = 1 vessels)	1.469	1.195	1.806	0.933	0.863	1.008	1.473	1.201	1.808
Comorbidity index (ref. = 2+)	0.517	0.428	0.623	1.085	1.015	1.160	0.520	0.432	0.626

HMI: High or middle-high income; LMI: Low or middle-low income

C.I.: confidence interval; LCL: Lower confidence limit; UCL: Upper confidence limit

The results of sensitivity analysis also showed that rural dwelling patients were less likely to select better patterns of healthcare provider selection (aOR = 0.635, 95% CI = 0.563–0.716/β = -0.455, standard error = 0.061; aOR = 0.820, 95% CI = 0.704–0.955/β = -0.199, standard error = 0.078), and the rural dwelling patients who selected worse patterns of healthcare provider selection had higher mortality risk (aOR = 1.403, 95% CI = 1.028–1.915/ β = 0.339, standard error = 0.158; aOR = 1.767, 95% CI = 1.188–2.627/ β = 0.569, standard error = 0.202). The results of Sobel’s test also validated that the mediation effects still existed. (Table 7).

**Table 7:** Results of Sensitivity Analysis.

	Model 1			Model 2			Model 3		
	Odds ratio	95% C.I.		Odds ratio	95% C.I.		Odds ratio	95% C.I.	
		LCL	UCL		LCL	UCL		LCL	UCL
Residential Area (ref. = Urban/ HMI)									
Rural/ LMI	1.512	1.104	2.072	0.635	0.563	0.716	1.403	1.028	1.915
Urban/ LMI	1.154	0.831	1.605	1.021	0.908	1.149	1.147	0.829	1.586
Rural/ HMI	1.826	1.221	2.730	0.820	0.704	0.955	1.767	1.188	2.667
Patterns of healthcare provider selection (ref. = excellent/short)							0.862	0.812	0.916

HMI: High or middle-high income; LMI: Low or middle-low income

Adjusted by hospital accreditation status, ownership, surgeon’s age, patient’s gender, age, number of vessels obstructed, and comorbidity index.

C.I.: confidence interval; LCL: Lower confidence limit; UCL: Upper confidence limit

## Discussion

Health is a natural right, and every government should provide sufficient and quality healthcare services for their people, and eliminate health disparities as much as possible. The issue of health inequity has long been studied; traditionally, researchers focused on accessibility of minor/disadvantage groups. In recent years, some discussions about eliminating health disparity have begun to advocate not only enhancing accessibility, but also improving quality of healthcare among minority groups to achieve health equality. [21–23] Recent studies have shifted their focus to explore whether inequity of quality of care exists among different patient characteristics, [24–26]; therefore, it is necessary to combine these two components together, when discussing the issue of health disparities, and it is also important to understand the patterns of healthcare provider selection under different settings.

Therefore, the current study not only discussed accessibility, but also the level of provider performance. Furthermore, the travelling distance to hospital was also taken into account. It is a novel perspective of health inequity studies, and it provides a multidimensional point of view. The results showed that rural patients with lower-income were prone to receive care from providers with poorer performance, compared with higher-income patients in urban areas. The travelling distance was varied among urban and rural dwelling patients with different income. Compared with higher-income patients in urban areas, the travelling distance of rural dwelling patients was longer, and urban dwelling patients with lower income were prone to stay in local areas to receive care. The results also revealed that relationships between urban and rural dwelling patients with different income levels and mortality were partially mediated by healthcare provider selection.

The island of Taiwan is shaped like a leaf that is narrow at both ends and is mountainous. The terrain in Taiwan is divided into two parts: the flat to gently rolling plains in the west, and the mostly rugged forest-covered mountains in the east. Ninety percent of the population in Taiwan lives in the west coastal plain, and most hospitals are also located in this area. Because the island is not too large, the travelling distance ought not to be a problem. An important surgery such as CABG must be undertaken in hospitals instead of clinics. Healthcare resources are not distributed equally, and most hospitals in Taiwan are located in or near to cities, especially medical centers. It might explain why rural dwelling patients move farther than do urban dwelling patients.

Nevertheless, why are urban patients with lower-income level prone to receive their care in local hospitals? Why do urban patients with higher income select hospitals farther away? This phenomenon might be illustrative from two perspectives. Firstly, although the NHI reduced the economic barrier of healthcare, the barriers of family care still exist. Poorer households have more financial constraints than do richer households. Therefore, if a family member needs to receive surgery, he/ she would be cared for by other family members, rather than hiring a nurse aide. However, because of the economic status, it is also not easy to take work leave for taking care of family members; therefore, it might be more convenient for a family member to provide the caregiving if a local or nearby hospital is selected to receive surgery. Furthermore, the findings of this study supported the notion that patterns of healthcare provider selection might cause the difference of mortality between urban and rural dwelling patients with different income levels, which meant the quality of care and travelling distances played a part in CABG surgery. It also implied that regionalization or decentralization of healthcare resources should be rethought. Regionalized vis-à-vis decentralized resources have a long historical struggle debate [27], with many pros and cons in each model from different dimensions. [28–30] Since travelling distance is still a concern for some patients, health authorities can think about the feasibility of regionalization, and allow a certain number of surgeons and hospitals in each healthcare service area to have the privilege of providing such surgeries.

The second perspective was the ability to select a better provider; we named this heath literacy in quality of care. Income level is usually used as one of the indicators of socioeconomic status, patients with higher socioeconomic status may select a better provider, [31] they may have better knowledge or be referred by friends or colleagues and so on.

How to decrease this kind of information asymmetry is an issue worth discussing. Prior literature provides evidence that information asymmetry may also result in the difference in choosing medical services, especially in rural and urban dwelling patients. [32] Taiwan is a highly information-based society, where all kinds of information can spread rapidly via e-mail, web community, and the internet and so on. However, the gap between urban and rural areas still exists. The degree of information spread in rural areas is lower than urban areas. Apart from the lack of infrastructure, rural dwellers’ characteristics themselves also form obstacles. Insufficient information might cause patients to select poorly performing surgeons/ hospitals. For decreasing this gap, health authorities should provide guidance (e.g. report card) to help patients select the optimal provider to receive their surgeries.

There are still three limitations that should be addressed.

1. The cutoff value of income level: The premium of Taiwan NHI is based on insured monthly salary.[8] Existing studies that have used the NHIRD to discuss the health inequality issues on income level in Taiwan usually employed monthly insured level as the basis for classification, and classified them. However, some employers do not buy insurance for their workers based on their real salary levels. In this study, our categorization of income level is more elaborate. The cutoff points are more reasonable than previous studies.
2. Calculation of travelling distance: Although understanding how far a patient moves to receive care is an interesting issue, we could not obtain the actual travelling distance from the existing literature and other sources. Using GIS software to calculate the distance between patient and hospital is one of the significant contributions of this study. However, patient’s ID and hospital’s ID were de-identified in Taiwan NHIRD; we also could not retrieve the addresses from this information. Using the coordinates of the center of town where hospitals were located in or where patients reside should be the optimal approach in this study; however, bias was still unavoidable.
3. Un-measurable variables. Although the study used the number of vessels obstructed and the comorbidity index as proxy indicators for disease severity and health status, other variables such as body mass index (which can also affect mortality), duration of operation, and level of blood sugar were not collected.

## Conclusions

Health disparity issues have long been recognized throughout the world. However, there is a lack of studies that examine how patterns of healthcare provider selection affect disparity in healthcare outcomes between patients of rural and urban areas with different income level. The findings of this study showed the patterns of healthcare provider selection varied among urban and rural dwelling patients with different income and impacted the relationships between urban and rural dwelling patients with different income level and mortality. The findings of this study could serve as a valuable reference for health policymaking to improve the public’s health.

## References

[1] Mensah GA. Eliminating disparities in cardiovascular health: six strategic imperatives and a framework for action. Circulation. 2005;111(10):1332–6. 15769777 10.1161/01.CIR.0000158134.24860.91

[2] Hsu CC, Lee CH, Wahlqvist ML, Huang HL, Chang HY, Chen L, et al Poverty increases type 2 diabetes incidence and inequality of care despite universal health coverage. Diabetes care. 2012;35(11):2286–92. 10.2337/dc11-2052 22912425 PMC3476930

[3] Shadmi E, Balicer RD, Kinder K, Abrams C, Weiner JP. Assessing socioeconomic health care utilization inequity in Israel: impact of alternative approaches to morbidity adjustment. BMC public health. 2011;11. 10.1186/1471-2458-11-609 PMC3171367 21801459

[4] Lin YH, Tseng YH, Chen YC, Lin MH, Chou LF, Chen TJ, et al The rural—urban divide in ambulatory care of gastrointestinal diseases in Taiwan. BMC international health and human rights. 2013;13. 10.1186/1472-698X-13-15 PMC3599496 23497027

[5] Masuda JR, Teelucksingh C, Zupancic T, Crabtree A, Haber R, Skinner E, et al Out of our inner city backyards: Re-scaling urban environmental health inequity assessment. Social science & medicine. 2012;75(7):1244–53. 22749441 10.1016/j.socscimed.2012.04.034

[6] Yu TH, Hou YC, Chung KP. Do low-income coronary artery bypass surgery patients have equal opportunity to access excellent quality of care and enjoy good outcome in Taiwan? International journal for equity in health. 2014;13(1):64. 25052723 10.1186/s12939-014-0064-8 PMC4159514

[7] Yu TH, Hou YC, Tung YC, Chung KP. Why do outcomes of CABG care vary between urban and rural areas in Taiwan? A perspective from quality of care. International journal for quality in health care: journal of the International Society for Quality in Health Care / ISQua. 2015. 10.1093/intqhc/mzv050 26239475

[8] Lu JFR, Hsiao WC. Does universal health insurance make health care unaffordable? Lessons from Taiwan. Health Affair. 2003;22(3):77–88. 10.1377/hlthaff.22.3.77 12757274

[9] Cheng TM. Reflections on the 20th anniversary of Taiwan's single-payer National Health Insurance System. Health Aff (Millwood). 2015;34(3):502–10. 25732502 10.1377/hlthaff.2014.1332

[10] Wen CP, Tsai SP, Chung WSI. A 10-year experience with universal health insurance in Taiwan: Measuring changes in health and health disparity. Ann Intern Med. 2008;148(4):258–67. 18283203 10.7326/0003-4819-148-4-200802190-00004

[11] Finks JF, Osborne NH, Birkmeyer JD. Trends in hospital volume and operative mortality for high-risk surgery. N Engl J Med. 2011;364(22):2128–37. 10.1056/NEJMsa1010705 21631325 PMC3150488

[12] Lien HM, Chou SY, Liu JT. Hospital ownership and performance: evidence from stroke and cardiac treatment in Taiwan. J Health Econ. 2008;27(5):1208–23. 10.1016/j.jhealeco.2008.03.002 18486978

[13] Tung YC, Chang GM, Chien KL, Tu YK. The relationships among physician and hospital volume, processes, and outcomes of care for acute myocardial infarction. Medical care. 2014;52(6):519–27. 10.1097/MLR.0000000000000132 24783991

[14] Chang HY, Bodycombe DP, Huang WF, Weiner JP. Risk-Adjusted Resource Allocation: Using Taiwan's National Health Insurance as an Example. Asia-Pac J Public He. 2015;27(2):Np958–Np71. 10.1177/1010539512471073 23343643

[15] Lin YH, Chen YC, Tseng YH, Lin MH, Hwang SJ, Chen TJ, et al Trend of urban-rural disparities in hospice utilization in Taiwan. PloS one. 2013;8(4):e62492 10.1371/journal.pone.0062492 23658633 PMC3637250

[16] Wu XD, Kumar V, Quinlan JR, Ghosh J, Yang Q, Motoda H, et al Top 10 algorithms in data mining. Knowl Inf Syst. 2008;14(1):1–37.

[17] Mangram AJ, Horan TC, Pearson ML, Silver LC, Jarvis WR. Guideline for Prevention of Surgical Site Infection, 1999. Infect Control Hosp Epidemiol. 1999;20(4):250–78. 10219875 10.1086/501620

[18] Baron RM, Kenny DA. The Moderator Mediator Variable Distinction in Social Psychological-Research—Conceptual, Strategic, and Statistical Considerations. J Pers Soc Psychol. 1986;51(6):1173–82. 3806354 10.1037//0022-3514.51.6.1173

[19] Mathieu JE, Taylor SR. A framework for testing meso-mediational relationships in organizational behavior. J Organ Behav. 2007;28(2):141–72.

[20] Zhang Z, Zyphur MJ, Preacher KJ. Testing Multilevel Mediation Using Hierarchical Linear Models Problems and Solutions. Organ Res Methods. 2009;12(4):695–719.

[21] Scott KW, Jha AK. Putting quality on the global health agenda. The New England journal of medicine. 2014;371(1):3–5. 10.1056/NEJMp1402157 24988552

[22] Beal AC. High-quality health care: the essential route to eliminating disparities and achieving health equity. Health Aff (Millwood). 2011;30(10):1868–71. 21976328 10.1377/hlthaff.2011.0976

[23] Kiguli J, Ekirapa-Kiracho E, Okui O, Mutebi A, Macgregor H, Pariyo GW. Increasing access to quality health care for the poor: Community perceptions on quality care in Uganda. Patient preference and adherence. 2009;3:77–85. 19936148 10.2147/ppa.s4091 PMC2778436

[24] Phipps MS, Jia H, Chumbler NR, Li X, Castro JG, Myers J, et al Rural-urban differences in inpatient quality of care in US Veterans with ischemic stroke. The Journal of rural health: official journal of the American Rural Health Association and the National Rural Health Care Association. 2014;30(1):1–6. 10.1111/jrh.12029 24383479

[25] Rumball-Smith J, Sarfati D, Hider P, Blakely T. Ethnic disparities in the quality of hospital care in New Zealand, as measured by 30-day rate of unplanned readmission/death. International journal for quality in health care: journal of the International Society for Quality in Health Care / ISQua. 2013;25(3):248–54. 10.1093/intqhc/mzt012 23411833

[26] Hsiao YY, Cheng SH. Is there a disparity in the hospital care received under a universal health insurance program in Taiwan? International journal for quality in health care: journal of the International Society for Quality in Health Care / ISQua. 2013;25(3):232–8. 10.1093/intqhc/mzt029 23548442

[27] Luft HS, Bunker JP, Enthoven AC. Should operations be regionalized? The empirical relation between surgical volume and mortality. The New England journal of medicine. 1979;301(25):1364–9. 503167 10.1056/NEJM197912203012503

[28] Halm EA, Lee C, Chassin MR. Is volume related to outcome in health care? A systematic review and methodologic critique of the literature. Ann Intern Med. 2002;137(6):511–20. 12230353 10.7326/0003-4819-137-6-200209170-00012

[29] Chowdhury MM, Dagash H, Pierro A. A systematic review of the impact of volume of surgery and specialization on patient outcome. The British journal of surgery. 2007;94(2):145–61. 17256810 10.1002/bjs.5714

[30] Singh JM, MacDonald RD. Pro/con debate: Do the benefits of regionalized critical care delivery outweigh the risks of interfacility patient transport? Critical Care. 2009;13(4):219–. 10.1186/cc7883 19678918 PMC2750128

[31] Zhang W, Ayanian JZ, Zaslavsky AM. Patient characteristics and hospital quality for colorectal cancer surgery. Int J Qual Health C. 2007;19(1):11–20. 10.1093/intqhc/mzl047 17000710

[32] McInnes DK, Gifford AL, Kazis LE, Wagner TH. Disparities in health-related internet use by US veterans: results from a national survey. Informatics in primary care. 2010;18(1):59–68. 20429979 10.14236/jhi.v18i1.754

